# Analysis of correlation of pre-therapeutic assessment and the final diagnosis in endometrial cancer: role of tumor volume in the magnetic resonance imaging

**DOI:** 10.3389/fonc.2023.1219818

**Published:** 2023-08-16

**Authors:** Elga López-González, Rocío García-Jiménez, Alberto Rodríguez-Jiménez, José Antonio Rojas-Luna, Cinta Daza-Manzano, Juan Gómez-Salgado, Rosa María Álvarez

**Affiliations:** ^1^ Gynaecological Oncology Unit, Department of Obstetrics and Gynaecology, Hospital Universitario Juan Ramón Jiménez, Huelva, Spain; ^2^ Department of Radiology, Hospital Universitario Juan Ramón Jiménez, Huelva, Spain; ^3^ Department of Sociology, Social Work and Public Health, Faculty of Labor Sciences, University of Huelva, Huelva, Spain; ^4^ Safety and Health Postgraduate Program, Universidad Espíritu Santo, Guayaquil, Ecuador; ^5^ Gynecological Oncology and Breast Cancer Unit, Department of Obstetrics and Gynecology, Hospital Universitario Santa Cristina, Madrid, Spain

**Keywords:** endometrial cancer, tumor volume, biopsy, magnetic resonance imaging, preoperative staging

## Abstract

**Objective:**

To evaluate whether the introduction of tumor volume as new parameter in the MRI assessment could improve both concordance between preoperative and postoperative staging, and the identification of histological findings.

**Methods:**

A retrospective observational study with 127 patients with endometrial cancer (EC) identified between 2016 and 2021 at the Juan Ramon Jimenez University Hospital, Huelva (Spain) was carried out. Tumor volume was measured in three ways. Analyses of Receiver Operating Characteristic (ROC) curve and the area under the curve (AUC) were performed.

**Results:**

Although preoperative MRI had an 89.6% and 66.7% sensitivity for the detection of deep mucosal invasion and cervical stroma infiltration, preoperative assessment had an intraclass correlation coefficient of 0.517, underestimating tumor final stage in 12.6% of cases, with a poor agreement between preoperative MRI and postoperative staging (κ=0.082) and low sensitivity (14.3%) for serosa infiltration. The cut-off values for all three volume parameters had good/excellent AUC (0.73-0.85), with high sensitivity (70-83%) and specificity (64-84%) values for all histopathological variables. Excellent/good agreement was found all volume parameters for the identification of deep myometrial invasion (0.71), cervical stroma infiltration (0.80), serosa infiltration (0.81), and lymph node metastases (0.81).

**Conclusion:**

Tumor volume measurements have good predictive capacity to detect histopathological findings that affect final tumor staging and might play a crucial role in the preoperative assessment of patients with endometrial cancer in the future.

## 1 Introduction

EC is the most common gynecological cancer in developed countries, with an increasing incidence rate over the past decades (1, 2). The overall five-year survival rate for initial stages ranges between 80-85% (2), and the prognosis depends on both an early diagnosis and an optimal management (3). An adequate pre-surgical assessment is one of the most important aspects in this regard. Preoperative evaluation based on endometrial biopsy and Magnetic Resonance Imaging (MRI) allows to assess whether the patient is an eligible candidate for surgery and to adjust its radicality as well (3). Attending to international recommendations, the standard surgical procedure for EC is a total hysterectomy and bilateral salpingo-oophorectomy, with the indication of lymphadenectomy being determined by several features such as tumor grade and uterine disease according to the FIGO stage (3). Although the Federation International of Gynecology and Obstetrics (FIGO) criteria recommends surgical staging for EC, pre-surgical assessment can limit the extent of this procedure, based on the probability of lymph node involvement and the risk of recurrence (4).

A problem that arises regarding the management of EC is the discrepancy between the preoperative test and the final postoperative diagnosis. For instance, the concordance rate for histology grade ranges from 60% to 80% (5–12). The largest published series including 1.804 patients found that the concordance rates for EC grades 1 2, and 3, were 75%, 53%, and 52%, respectively. Regarding imaging testing, there is an underestimation of myometrial involvement above 50% in 21-25% of cases (13, 14). Furthermore, the substantial lymph-vascular space invasion (LVSI) status rarely matches that of the preoperatory biopsy. Thus, correct pre-surgical evaluation is essential to avoid re-interventions or unnecessary lymphadenectomies, which increase surgical morbidity (15), as well as underdiagnosis which might increase recurrence and morbidity rates.

Therefore, our main objective was to evaluate whether the introduction of tumor volume as new parameter in the MRI assessment could improve both concordance between preoperative and postoperative staging, and the identification of histological findings, based on the histopathological examination of the surgical specimen as a *gold standard*. As a secondary objective, we wanted to identify variables which might be associated with a higher concordance between the preoperative and postoperative staging.

## 2 Methods

An observational, retrospective, cross-sectional study was conducted between January 2016 and December 2021, including 194 patients who had been diagnosed with endometrial cancer at the Juan Ramon Jimenez University Hospital, Huelva. The study received the approval of the local Institutional Review Board (2534-N-21), and it was carried out following the ethical principles of the Declaration of Helsinki.

**Figure 1:**
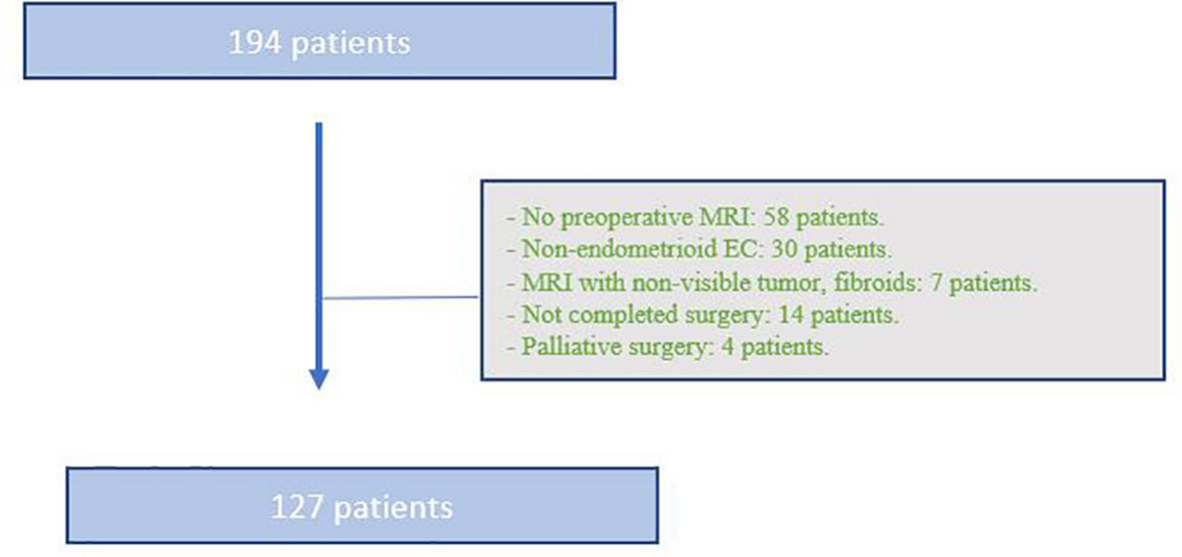
Flow chart of patients (Tumor volume in Endometrial cancer, Spain, 2022).

Patients were retrospective and consecutively recruited from a prospective database provided by the gynecologic oncology unit at the Juan Ramon Jimenez University Hospital. All participants gave their informed consent to access their clinical data. The inclusion criteria were patients with a histological diagnosis of EC who underwent complete surgical staging. The exclusion criteria were non-Endometroid EC, patients who refused surgery, patients who did not underwent MRI assessment or without visible tumor, and those who received neoadjuvant therapy (**Figure 1**).

### 2.1 Preoperative staging

The initial preoperative diagnosis was made after endometrial sampling, taken either with hysteroscopy or a Pipelle device, reporting tumor grade and histologic type. All included patients in our study underwent MRI as part of preoperative staging. Preoperative pelvic MRIs were performed on equipment with a 1.5T magnetic field (Phillips Healthcare or General Electric Medical Systems). High-resolution T2-weighted turbo spin-echo sequences were performed (3 mm slice thickness) in the three orthogonal or oblique planes appropriate to the major and minor axes of the body of the uterus, as well as diffusion-weighted planar echo sequence (DWI) with a maximum b value > 600 sec/mm2 and corresponding apparent diffusion coefficient (ADC) maps. Additional sequences (weighted on T1 or T2 with fat saturation) as well as the use of T1 sequences with fat saturation after administration of gadolinium were used at the discretion of the supervising radiologist of the study. MRIs were analyzed by expert radiologists with more than 10 years of experience in gynecological cancer images.

Standard imaging assessment included the evaluation of myometrial (less or more than 50%), cervical and serosa invasion, as well as lymph node metastases. Staging was performed according to the FIGO classification. Tumor volume was introduced as an additional parameter, which was measured in three ways. The maximum diameters of both the tumor and the uterus were measured by adopting three mutually perpendicular distances, taking the largest diameter as a reference. The estimated volumes of the neoplasm and the uterus were calculated by means of an ellipsoid formula [Longitudinal x Transverse x Anteroposterior x (π/3)]. The neoplasm/uterus volume ratio (N/U) was also calculated. Subsequently, tumor volumetry was assessed by manual contouring of the lesions, a freehand ROI (region of interest), in which the neoplasm showed the largest size, usually in T2W. The volume data was evaluated using the standard software (LiveWire, Carestream or Phillips VUE PACS tool).

### 2.2 Postoperative staging

Following the FIGO classification, depending on the initial endometrial biopsy and the preoperative imaging, patients were stratified in recurrence risk groups (16). Those who were candidates for surgery, underwent total hysterectomy and bilateral salpingo-oophorectomy (HT), with pelvic or pelvic and para-aortic lymphadenectomy in the group risk for node metastases. After surgery, surgical specimens were submitted for histopathological evaluation performed by expert pathologists with wide experience in gynecological cancers. Postoperative histological report included surgical findings such as tumor grade, histologic type, depth of invasion, lymphovascular space invasion, cervical and adnexal involvement, and nodal status. Histologic grade followed WHO Classification of Tumors (5th edition) (17). The depth of myometrial infiltration was divided in less or more than 50%. Adjuvant treatment was selected based on the FIGO stage, final type and tumor grade with the consensus of the oncology local committee.

### 2.3 Data collection

Demographic and clinical parameters collected were age at diagnosis, body mass index (BMI), menopausal status, parity, abnormal uterine bleeding, history of breast cancer, comorbidities (hypertension, dyslipidemia, and diabetes mellitus), family history of EC, surgical treatment (HT, HT and pelvic lymphadenectomy, or HT and pelvic and aortic lymphadenectomy), preoperative FIGO stage, and adjuvant treatment (chemotherapy, external radiation therapy, brachytherapy, or hormonal treatment).

Variables related to the preoperative assessment were deep myometrial invasion (defined as more than 50%), cervical stroma infiltration, serosa infiltration, lymph node metastases, preoperative FIGO stage. Postoperative staging variables were also collected, such as histopathological type and grade; myometrial, cervical and serosa invasion; lymph node metastases; and postoperative FIGO stage.

Finally, we collected the parameters related to the tumor volume as an additional new parameter to evaluate their capacity to predict tumor invasion. These parameters were tumor ellipse, neoplasm/uterus volume ratio, and ROI tumor volumetry (ROI).

### 2.4 Statistical analysis

The statistical analysis was carried out using the IBM SPSS software version 24.0 (SPSS Inc., Chicago, IL, USA). Quantitative variables were described as mean and standard deviation (SD) data, or median and interquartile range (IQR) for non-normally distributed data, while frequencies and percentages were used for qualitative variables. The normality of the data was assessed using the Kolmogorov-Smirnoff test. The association between qualitative variables was evaluated using the Chi-square test or Fisher exact test.

For our main objective, sensitivity and specificity were computed considering histopathological postoperative examination as the *gold standard*. The strength of association between preoperative and postoperative staging, as well as between the three tumor volume parameters, was assessed estimating either the Kappa statistic (18) or intraclass correlation coefficients (ICC) (19). Scores reflected agreement as: ≤0.2 poor, 0.21–0.4 fair, 0.41–0.60 moderate, 0.61–0.8 good/substantial, ≥0.81 excellent/near perfect. Analyses of Receiver Operating Characteristic (ROC) curve and the area under the curve (AUC) were performed to evaluate the diagnostic capability of the tumor volume parameters, as well as to assess the optimal cut-off value for each parameter.

For our second objective, we performed a secondary analysis to evaluate the variables which might be associated with a higher concordance between the preoperative and postoperative staging. For this purpose, the sample size was divided in two groups, depending on whether the preoperative and postoperative results were concordant or discordant. Tumor volume parameters, as well as clinical variables, were compared between groups.

## 3 Results

A total of 194 patients with endometrial cancer were identified over the period of study, out of which 67 were excluded. Thus, the final sample consisted of 127 patients who completed the study. Demographic and clinical variables are displayed in **Table 1**. As can be seen, the mean age at diagnosis was 63.4 years, with most of them (85.1%) presenting with abnormal uterine bleeding. The majority of patients were at postmenopausal stage (85.1%) and received adjuvant treatment (58.3%). Only patients with endometrioid histological subtype were included. They were distributed according to the degree of differentiation as follows: low grade (G1-G2) 106 patients (83.5%) and high grade (G3) 21 patients (16.5%). When analyzed by age, patients older than 65 years (25.5%) have a higher proportion of high degree of tumor differentiation compared to younger patients (9.7%), with a statistically significant difference (p: 0.018),

**Table 1:** Demographic and clinical variables (Tumor volume in Endometrial Cancer, Spain, 2022).

Variables	Median (range)/*n* (%)
Age at diagnosis	63.4 (28-91)
BMI	26.3 (15-36)
Postmenopausal Stage	102 (85.1%)
Parity	2.3 (0-9)
Abnormal uterine bleeding	79 (62.2%)
History of breast cancer	9 (7.1%)
Comorbidities	
Hypertension	77 (60.6%)
Dyslipidemia	41 (32.2%)
Diabetes mellitus	30 (23.6%)
Family history of EC	9 (7.08%)
Surgical Treatment	
HT	50 (39.4%)
HT + pelvic lymphadenectomy	51 (40.2%)
HT + pelvic and aortic lymphadenectomy	26 (20.5%)
Adjuvant treatment	74 (58.3%)
Chemotherapy	27 (36.4%)
External radiation therapy	3 (0.5%)
Brachytherapy	35 (47.3%)
Hormonal treatment	1 (1.3%)

BMI, Body mass index; HT, Hysterectomy + bilateral salpingo-oophorectomy.

The comparison between the preoperative and postoperative staging is shown in **Table 2**. The preoperative assessment underestimated and overestimated the tumor stage in 12.6% (*n*=16) and 2.36% (*n*=3) of cases, respectively. The stage was concordant in the two assessments in 81.89% (*n*=104) of cases, with an intraclass correlation coefficient of 0.517 (95% IC 0.377-0.634). The limited number of patients with stromal involvement could be a limitation of the study.

**Table 2:** Preoperative and postoperative staging (Tumor volume in Endometrial Cancer, Spain, 2022).

Stages	Preoperative staging	Postoperative staging	p	ICC	95% CI
I	108 (85.04%)	95 (74.8%)	<0.001	0.517	0.377 - 0.634
II	6 (4.72%)	1 (0.79%)			
III-IV	13 (10.24%)	31 (24.41%)			

Regarding the FIGO final stage, the distribution was as follows: 53 in FIGO stage IA (41%); 42 in FIGO stage IB (33.1%); 1 in FIGO stage II (0.8%); 28 in FIGO stage III (17.3%); 3 in FIGO stage IV (2.4%). The specific data referring to myometrial, cervical, and serosa invasion are listed in **Table 3**. In **Table 4** we can see the diagnostic accuracy of preoperative MRI regarding surgical findings after histopathological examination of surgical specimen. The preoperative MRI assessment of myometrial invasion and cervix infiltration was concordant in 87.4% and 96.9% of cases, respectively, with good agreement as shown by the Cohen’s kappa index of 0.74 and 0.65. However, although the concordance regarding serosa infiltration was of 84.3%, Cohen’s kappa index of 0.082 showed poor agreement, with low sensitivity (14.3%).

**Table 3:** Concordance Preoperative FIGO Stage (Tumor volume in Endometrial Cancer, Spain, 2022).

FIGO Stage	Final FIGO					
		IA	IB	II	III-IV	Total (*n*)
MRI	**IA**	47	2	0	6	55
	**IB**	6	38	0	8	52
	**II**	0	1	0	3	4
	**III-IV**	27	9	1	46	83

**Table 4:** Evaluation of the diagnostic accuracy of preoperative MRI for histological findings (Tumor volume in Endometrial Cancer, Spain, 2022).

Histological findings	Preoperative staging	Postoperative staging	Concordance	Sensitivity	Specificity	*κ* value95% CI
Deep myometrial invasion	69 (54.3%)	67 (52.8%)	87.4%	89.6%	85.0%	0.74 (0.63-0.86)
Cervical stroma infiltration	6 (4.7%)	6 (4.7%)	96.9%	66.7%	98.3%	0.65 (0.31-0.98)
Serosa infiltration	14 (7.9%)	14 (11.0%)	84.3%	14.3%	92.9%	0.082 (0.001-0.451)

**Figure 2:**
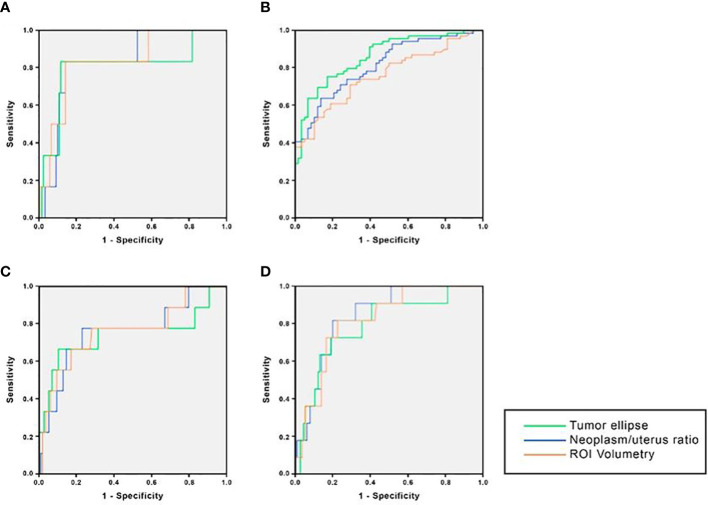
ROC curves for the prediction of histological findings of tumor volume parameters. **(A)** Deep myometrial invasion; **(B)** Cervical stroma infiltration; **(C)** Serosa infiltration; **(D)** Lymph nodes metastases (Tumor volume in Endometrial cancer, Spain, 2022).

We analyzed the parameters related to tumor volume and their capacity to predict postoperative histopathological findings. ROC curves were calculated for tumor ellipse, neoplasm/uterus volume ratio, and ROI volumetry, as displayed in **Figure 2**. Based on these ROC curves, cut-off values of these parameters were estimated to apply in the prediction of useful preoperative variables. The results are displayed in **Table 5**. As can be seen, most of the cut-off values found for all three volume parameters showed excellent predictive capability for histopathological findings, with high sensitivity and specificity.

**Table 5:** Evaluation of the predictive capacity of tumor volume parameters regarding histological findings (Tumor volume in Endometrial Cancer, Spain, 2022).

Tumor volume parameters Mean (Range)	Deep myometrial invasion	Cervical stroma infiltration	Serosa infiltration	Lymph nodes metastases
Tumor ellipse 24 (1.5-290)	9.01 cm3	25.46 cm3	24.89 cm3	26.00 cm3
	AUC: 0.77 p<0.0005	AUC: 0.83 p:0.006	AUC: 0.76 p:0.009	AUC: 0.84 p:0.0005
	Se: 73%; Sp: 70%	Se: 83%; Sp: 79%	Se: 78%; Sp: 78%	Se: 81%; Sp: 80%
Neoplasm/uterus ratio 0.21 (0.01-0.77)	0.11 cm3	0.36 cm3	0.21 cm3	0.30 cm3
	AUC: 0.85 p<0.0005	AUC: 0.80 p:0.013	AUC: 0.74 p:0.015	AUC: 0.79 p:0.001
	Se: 82%; Sp: 74%	Se: 83%; Sp: 84%	Se: 77%; Sp: 70%	Se: 72%; Sp: 80%
ROI volumetry 21 (1-278)	11.1 cm3	25.05 cm3	19.6 cm3	27.9 cm3
	AUC: 0.73 p<0.005	AUC: 0.83 p:0.006	AUC: 0.76 p:0.009	AUC: 0.82 p<0.0005
	Se: 70%; Sp: 64%	Se: 83%; Sp: 80%	Se: 78%; Sp: 72%	Se: 72%; Sp: 84%
*κ* value	0.71	0.80	0.81	0.81

AUC, area under the curve; Se, sensitivity; Sp, specificity.

The correlation between the different radiological tumor volume measurement parameters was analyzed. The Ellipse tumor parameter was positively related both to the Ratio parameter (Pearson coefficient= 0.673) with a moderate relationship and to the ROI parameter (CC= 0.917) with a strong relationship. Both were statistically significant (p= 0.000). The ROI parameter was positively related both to the Ratio parameter (Pearson coefficient= 0.608) with a moderate relationship and to the Ellipse parameter (CC= 0.917) with a strong relationship. Both were statistically significant (p= 0.000). In addition, a secondary analysis was carried out to evaluate which variables might be associated with a higher concordance between the preoperative and postoperative staging. The results are displayed in **Table 6**. We found no difference between discordant and concordant cases regarding age, BMI or postmenopausal stage. In contrast, we found that concordant cases had lower values than discordant cases for all tumor volume parameters: tumor ellipse (43.15 vs 20; p:0.038), neoplasm/uterine ratio (0.27 vs 0.19; p:0.037), and ROI volumetry (35.67 vs 18.1; p:0.031).

**Table 6:** Evaluation of variables associated with preoperative and postoperative concordance (Tumor volume in Endometrial Cancer, Spain, 2022).

Variables	Discordant cases	Concordant cases	p
Age at diagnosis	64 ± 10	63 ± 11	0.785
BMI	28 ± 5	30 ± 6	0.258
Postmenopausal stage	20 (87%)	88 (84.6%)	0.776
Tumor ellipse (cm^3^)	43.15 ± 72.2	20 ± 29.4	0.038
Neoplasm/Uterus ratio (cm^3^)	0.27 ± 0.2	0.19 ± 0.25	0.037
ROI Volumetry (cm^3^)	35.67 ± 57.5	18.1 ± 21.9	0.031
Tumor Grade G1-2	17 (73.9%)	89 (85.6%)	0.173
G3	6 (26.1%)	15 (14.4%)	

## 4 Discussion

According to international guidelines, preoperative evaluation based on endometrial biopsy and MRI assessment allows for an extension study and to assess whether the patient might be a candidate for surgery, adjusting its radicality (4). However, there is a long-going problem regarding the surgical management of EC, which is the discrepancy between preoperative evaluation and the postoperative diagnosis. Reports on sensitivity of preoperative MRI staging of EC reflect a great diversity, ranging from 37% to 79% (13, 14, 20, 21). Few studies specifically evaluated the accuracy of MRI to predict FIGO stage, describing concordance rates that vary from 33.1% to 86.3% (21, 22). For instance, the reported accuracy rates of MRI assessment of myometrial invasion ranges from 65-89% (13, 14, 20, 21), similarly to the 87.4% concordance rate found in our study. However, myometrial invasion shows more discrepancy in postmenopausal patients due to the thinning of myometrial tissue, while the concordance was higher for patients under sixty-years old (23). We evaluated factors that might affect the diagnosis according to previous publications (23), but we found that they did not affect our results. Our sample was homogenous, consisting of patients with endometroid EC, with 85% of them being postmenopausal with a similar age at diagnosis.

Our results showed that preoperative MRI assessment had an 89.6% and 66.7% sensitivity for the detection of deep myometrial invasion and cervical stroma infiltration, respectively, which is similar those previously published (20, 21). Nonetheless, in terms of detecting serosa infiltration, sensitivity dropped to 14%, with a poor agreement between preoperative MRI and postoperative staging (κ=0.082), far from quality standards. This caused an underestimation of the final FIGO stage in 12.6% of our patients (*n*=16), who required a second intervention to perform a lymphadenectomy to complete the surgical staging. This emphasizes the need for new preoperative tools to improve the pre-surgical classification of EC.

All these results seem to suggest that current preoperative tests do not reliably predict the final surgical findings, especially in initial tumor stages and our results agree with previous studies (13, 14, 21, 22), highlighting the need for new preoperative assessment tools. Although tumor size is one of the determining factors on disease staging for some gynecological cancers, such as cervical or breast cancer, this has not been the case for endometrial cancer (24). Ytre-Hauge S et al. (25) proposed a risk model using specific cut-off values of tumor size to predict histological findings. However, the conclusions made in their study are based in the use of diameters to measure tumor size. Although traditionally diameter has been used to assess tumor size (26), this is not always a reliable parameter, given its usually irregular and spheric shape (27). Furthermore, measurements based on volume have proven to be more reproducible (28, 29), which weakens the conclusions reached by previous studies based on diameter measurements.

Volume tumor has been described as a good predictor of final EC stage in several studies. The study published by Todo et al. (30) was one of the first to use the Ellipse formula for this purpose. However, their results were inconsistent, with low sensitivity and specificity, as well as high interobserver variability, thus failing to determine specific cut-off values to predict the final stage. In contrast, in our study we applied tumor volume to evaluate the prediction of histopathological findings measured with three different parameters: tumor ellipse, neoplasm/uterus ratio, and ROI volumetry. The cut-off values found for all three parameters had AUC ranging between 0.73-0.85, with high sensitivity and specificity values between 70-83% and 64-84%, respectively. Although the routine preoperative MRI and the tumor volume assessment had similar sensitivity (89.6% vs 70-82%), and specificity (85.0% vs 83%) values for the detection of deep myometrial invasion, the results of tumor volume showed a clear improvement in the case of the other histological variables, with a false negative (FN) rate around 20%. For the detection of cervical stroma infiltration, all three tumor volume parameters had higher sensitivity (66.7% vs 83%), with slightly lower specificity (98.3% vs 79-84%); with a FN rate around 17%. The clearest example of improvement of tumor volume assessment is in the case of serosa infiltration though, with a striking higher sensitivity (14.3% vs 77-78%, 22% of FN rate). In is also worth mentioning our results regarding detection of lymph nodes metastases, with an AUC of 0.84, 0.79, and 0.82, for tumor ellipse, neoplasm/uterus ratio and ROI volumetry, respectively, as well as a high sensitivity (72-81%, with 20-28% of FN rate) and specificity (80-84%) values. We also evaluated the agreement between the three volume parameters to identify the histopathological findings, with excellent agreement for the identification of cervical stroma infiltration (0.80), serosa infiltration (0.81), and lymph node metastases (0.81), and good agreement in the case of deep myometrial invasion (0.71), showing a consistent predictive capacity of tumor volume for all histopathological parameters.

Amongst the strengths of our study is its novelty and originally, given the introduction of a new parameter such as tumor volume measurement for the preoperative assessment of EC patients. To our knowledge, this is the first study to evaluate the predictive capacity of three different tumor volume measurements to predict postoperative surgical findings in patients with EC. Nonetheless, our study also has its limitations, with its retrospective nature being the main one. Another limitation of this study could be the exclusion of the sentinel lymph node as a study variable, since it was not studied in the entire population and this could be a source of bias. These limitations will be addressed in future prospective studies carried out on the EC population in our area. As established and recommended in our current guidelines (31), patients classified preoperatively as low and intermediate risk group will undergo Selective Sentinel Lymph Node Biopsy (SLNB) and lymphadenectomy can be omitted in these cases. The same does not occur in high-risk patients, where lymphadenectomy is necessary in the surgical staging. Again, the concordance of the pre-surgical imaging parameters, which correspond to a correct classification of the patients, is extremely important in this algorithm when introducing SLNB. Future prospective studies should evaluate the application of this new assessment tool to confirm its and validity and clinical usefulness.

All in all, this study constitutes an essential first step towards the implementation of tumor volume as part of the preoperative assessment of patients with endometrial cancer. Our results show that tumor volume measurements have good predictive capacity to detect histopathological findings that affect final tumor staging and might play a crucial role in the preoperative assessment of patients with endometrial cancer in the future.

## Data availability statement

The raw data supporting the conclusions of this article will be made available by the authors, without undue reservation.

## Ethics statement

The study received the approval of the Huelva Regional Review Board (2534-N-21). The patients/participants provided their written informed consent to participate in this study.

## Author contributions

All the authors have intellectually contributed to the work, met the conditions of authorship, and approved its final version. This work is original and has not been previously published and is not under review by any other journal. This manuscript conforms to the ICMJE Recommendations for the Conduct, Reporting, Editing, and Publication of Scholarly Work in Medical Journals. Conceptualization, ELG, ARJ, JARL, CDM, JGS, and RMA; Data curation, ELG, ARJ, JARL, and CDM. Formal analysis, ELG, ARJ, JARL, CDM, JGS and RMA; Investigation, ELG, ARJ, JARL, CDM, JGS and RMA; Methodology, ELG, ARJ, JARL, CDM, JGS and RMA; Project administration, ELG and RMA; Resources, ELG, ARJ, JARL, CDM, JGS and RMA; Software, ELG, ARJ, CDM, JGS and RMA; Supervision, ELG, JARL, JGS and RMA; Validation, ELG, ARJ, JARL, CDM, JGS and RMA; Visualization, ELG, ARJ, JARL, CDM, JGS and RMA; Writing – original draft, ELG, ARJ, JARL, CDM, JGS and RMA; Writing – review & editing, ELG, ARJ, JARL, CDM, JGS and RMA.
